# Ante-Natal and Intra-Natal Care

**Published:** 1919-07-26

**Authors:** 


### ANTE-NATAL AND INTRA-NATAL CARE.

In a thoughtful paper read on July 1, before the National Conference on Infant Welfare, organised by the National Association for the Prevention of Infant Mortality, Dr. Eardley Holland attempts to assess the exact value of ante-natal care, as far as that is practicable. It is too often, as he points out, assumed as axiomatic that by ante-natal care an enormous saving of fœtal life can be effected, and that the whole of such a saving will necessarily increase the birth-rate by that amount. He then draws attention, and not before it is time, to the limitations of what ante-natal care can do, both actually and relatively, to what he calls intra-natal care—that is, to improvements in the practice of obstetrics. After balancing as fairly as possible the various factors which cause still-births, he concludes that about 52 per cent. of the latter at the most are preventable; that of this proportion five-thirteenths are preventable by ante-natal care, five-thirteenths by intra-natal care, and the other three-thirteenths by a combination of the two. So that, useful and valuable as ante-natal care undoubtedly is, it is only equal, and not superior, to intra-natal care as a preventive of fœtal death. It is therefore necessary that ante-natal care shall not be allowed to monopolise the attention of those who seek to diminish infant mortality.

Further, Dr. Holland points out that when all fœtal deaths that can be prevented *are* prevented, a birth-rate of 20 per 1,000 will be raised only to 20.3, an improvement which must certainly be striven for, but in itself is a small factor in the problems of population and of race suicide. He also quite properly insists that when this saving of infant life is brought about it by no means follows that the population will be correspondingly increased. "The birth-rate," he says, "is governed by profound economic factors beyond the influence of obstetrical science. If obstetrical science could bring it about that every conception reached healthy maturity the result would soon be a reduction in the number of conceptions." This warning, sound as it certainly seems to be, is naturally not intended by Dr. Holland as a discouragement to infant welfare work, but only as a contribution to the study of the subject, and as a prevention of disappointment through pitching expectation too high. His figures and statistics appear open to one objection, however, in that they are based on the causes of and figures relating to still-birth, without taking into account those of abortion. If the latter were included it would almost certainly result in a proportionate increase of the preventable deaths credited to ante-natal as opposed to intra-natal care. This, of course, by no means invalidates his conclusions against too hasty generalisations as to results, or his plea for the due development of intra-natal care along with the ante-natal work. Altogether his contribution is a much-needed spur to clear thinking on this important question, and should be studied by all who are interested in this rapidly growing subject.

